# Chemotherapy for locoregionally advanced nasopharyngeal carcinoma: Who really needs it

**DOI:** 10.1002/cam4.5497

**Published:** 2022-12-09

**Authors:** Weiling Qu, Xuan Wang, Qiao Qiao, Yanli Wang

**Affiliations:** ^1^ Department of Radiation Oncology the First Hospital of China Medical University Shenyang Liaoning China; ^2^ Department of Radiation Oncology, Women's Hospital, School of Medicine Zhejiang University Hangzhou Zhejiang China

**Keywords:** chemotherapy, locoregional advanced, nasopharyngeal carcinoma, node‐negative, SEER

## Abstract

**Objectives:**

The CSCO and ASCO guidelines in 2021 recommend chemotherapy for stage III–IVA (8th edition of AJCC staging) nasopharyngeal carcinoma (NPC). Actually, patients with stage T3–4N0M0 are often excluded from various clinical trials of the locoregionally advanced NPC, and the survival benefit of chemotherapy in such patients has always been controversial. This study aims to explore the benefit of chemotherapy in patients with locoregionally advanced NPC, especially those with negative lymph nodes.

**Methods:**

A total of 2741 patients were extracted from the SEER database. After a 1:1 PSM analysis, 272 patients were obtained to further explore whether the addition of chemotherapy would achieve survival benefits.

**Results:**

After PSM, Kaplan–Meier curves showed that the overall survival (OS) of patients receiving chemoradiotherapy (*p* = 0.031) was higher than those receiving radiotherapy alone. Similar results were observed for cancer‐specific survival (CSS). We further stratified the patients according to lymph node status and found that the addition of chemotherapy in patients with positive lymph nodes could significantly improve 5‐year OS rates (58.08% vs. 43.95%; *p* = 0.025) and 5‐year CSS rates (67.42% vs. 51.95%; *p* = 0.015) compared with radiotherapy alone, but there was no additional benefit of chemotherapy in patients with negative lymph nodes. For all 449 cases of T3–4N0M0 NPC, radiotherapy improved the OS rates (HR 0.293, 95% CI 0.203–0.424) and the CSS rates (HR 0.252, 95% CI 0.171–0.371) compared with no radiotherapy, while chemotherapy did not show significant survival benefit compared with no chemotherapy.

**Conclusion:**

Our results reveal that stage T3–4N0M0 NPC may be exempted from chemotherapy, and use radiotherapy alone to reduce toxic and side effects. These results still need to be verified by future prospective trials.

## 1 INTRODUCTION

Nasopharyngeal carcinoma (NPC) is a malignant tumor with geographic characteristics. It has a low incidence in most of the world, but it is more common in southern China and Southeast Asia.^1^ According to the data of GLOBOCAN in 2020, there were 133,354 new cases of NPC and 80,008 deaths worldwide. Among them, 62,400 cases of NPC in China were newly diagnosed and 34,800 cases died. As a malignant tumor with geographic characteristics in China, Chinese NPC patients account for nearly 50% of global patients.^2^ Due to the sensitivity of NPC to radiotherapy and chemotherapy and the special anatomical position of the nasopharynx, radiotherapy and chemotherapy are currently the main treatment methods of NPC.^3^ With the emergence and development of intensity‐modulated radiation therapy (IMRT), the curative effect of NPC has been significantly improved.^4^

For stage I NPC, radiotherapy alone is currently the main treatment option.^5^ For locoregionally advanced NPC, there have been many researches that have proved that radiotherapy combined chemotherapy treatments can improve the survival rate and local control rate, such as induction chemotherapy (IC), concurrent chemotherapy, adjuvant chemotherapy (AC).^6^, ^7^, ^8^, ^9^, ^10^ The national comprehensive cancer network (NCCN) guidelines have proposed the use of concurrent chemoradiotherapy (CCRT) combined with AC or IC followed by CCRT as a level 2A recommendation, and CCRT alone as a level 2B recommendation for stage II–IVA (8th edition of AJCC staging) NPC.^11^ However, accurate radiotherapy planning and coordination between radiotherapy and chemotherapy are critical to the prognosis of patients. Encouragingly, the newly released Chinese Society of Clinical Oncology (CSCO) and American Society of Clinical Oncology (ASCO) guidelines in 2021 address outstanding and important clinical problems in radiotherapy and chemotherapy for stage II–IVA NPC. Among them, CCRT+AC or IC + CCRT are recommended for patients with stage III–IVA NPC (except T3N0), while for T3N0 patients, CCRT is recommended. AC or IC can also be used.^12^

However, we have noticed that some clinical studies for locoregionally advanced NPC have excluded patients with negative lymph nodes, such as the researches of some large‐scale phase 3 clinical trials of Sun Yat‐sen University cancer center.^13^, ^14^, ^15^ Additionally, some retrospective studies found that in patients with locoregionally advanced nasopharyngeal carcinoma, there was no significant difference in overall survival (OS), local recurrence‐free survival (LRFS), and disease‐free survival (DFS) between the two groups who received CCRT and those who receive radiotherapy alone.^16^, ^17^, ^18^ Moreover, whether it is two‐dimensional radiotherapy or intensity‐modulated radiotherapy, several prospective randomized controlled studies have shown that the addition of concurrent chemotherapy significantly increases the acute toxicity of patients during radiotherapy.^19^, ^20^, ^21^

In conclusion, for patients with locoregionally advanced and low‐risk NPC, the value of combined chemotherapy is challenged. Therefore, this study intends to explore the therapeutic efficacy and prognostic factors of locoregionally advanced NPC based on the Surveillance, Epidemiology, and End Results (SEER) database, especially pay attention to the survival benefits of chemotherapy based on radiotherapy for patients with negative lymph nodes, hoping to provide a certain basis for clinical practice and clinical trials in the future.

## 2 MATERIAL AND METHODS

### 2.1 Study population

We performed a retrospective study of locoregionally advanced NPC patients by using data from the National Cancer Institute SEER program (https://seer.cancer.gov/data/). The seer database surveys approximately 27.8% of the United States population, which represent the overall level of the United States in terms of demographics, cancer incidence, and mortality.

The inclusion criteria included: (1) International Classification of Disease‐O‐3 (ICD‐O‐3) site codes C11.0‐C11.3, C11.8, and C11.9 and histologic codes 8010, 8020, 8021, 8070–8073, 8082, and 8083; (2) NPC diagnosed as stage III–IVB according to the 6th and 7th editions of the American Joint Commission on Cancer (AJCC) staging system, where stage IVB refers to any T, N3, M0; (3) patients had positive histological findings; (4) NPC was the first and only primary malignancy. We also excluded the following patients: (1) survival data were missing or unknown; (2) reporting source was autopsy or death certificate only. Finally, a total of 2741 eligible patients from 2004 to 2016 were enrolled in our study.

### 2.2 Variable definitions

The demographic variables of the patients were obtained from the database, including age at diagnosis, sex, race, and marital status. The tumor characteristics were extracted from the database, including grade, histology, stage, T stage, and N stage. Also the treatment methods, such as surgery to the primary site, radiotherapy, and chemotherapy, were also collected. According to the World Health Organization (WHO) classification scheme, we divided the histology of NPC into four categories, of which ICD‐O‐3 codes 8070 and 8071 were keratinizing squamous cell carcinoma (KSCC), ICD‐O‐3 codes 8072 and 8073 were differentiated non‐keratinizing squamous cell carcinoma (DNKSCC), ICD‐O‐3 codes 8020, 8021, 8082, and 8083 were undifferentiated non‐keratinizing squamous cell carcinoma (UNKSCC), and ICD‐O‐3 code 8010 were classified as other unspecified Group. OS status and cancer‐specific survival (CSS) status were also collected as the outcomes of our study.

### 2.3 Statistical analysis

Descriptive analysis was used for each demographic and clinicopathological variable. Student's *t*‐test or independent‐samples Mann–Whitney *U*‐test was applied to the continuous variable. Pearson's chi‐squared test or Fisher's exact test of categorical variables was used to compare the differences in covariate distributions between patients who received radiotherapy only and those who received chemoradiotherapy. The 1:1 paired propensity score matching (PSM) was performed to balance the observed bias in baseline characteristics between the two treatment groups. The standard deviation of each covariate was applied to compare the similarity between the two groups, and the absolute value less than 0.1 represented a good balance. Independent prognostic indicators were evaluated by univariate and multivariate Cox analysis. Kaplan–Meier curves and log‐rank (Mantel–Cox) tests were applied to describe the survival rates of patients. We use SPSS 23.0 (SPSS, Chicago), GraphPad Prism 8 (GraphPad Software), and R software (version 4.2.1) for all statistical analysis, and two‐tailed tests and *p*‐values <0.05 is considered statistically significant in all statistical analyses.

## 3 RESULTS

### 3.1 Patient characteristics

**FIGURE 1:**
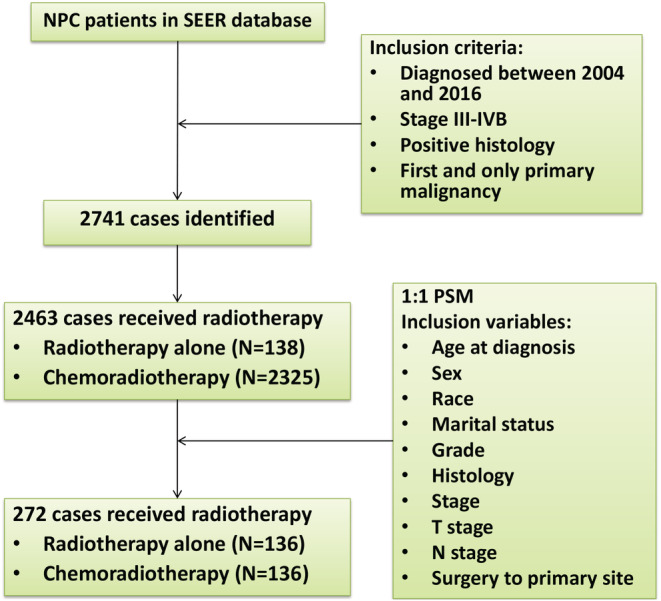
Flowchart of patient selection in this study

We included a total of 2741 patients with stage III–IVB NPC from the SEER database (Figure 1), of which 1388 (50.6%) were diagnosed with stage III, 863 (31.5%) were diagnosed with stage IVA, and 490 (17.9%) were diagnosed with stage IVB. The baseline characteristics of all patients were listed in Table S1. The median age was 53 years (interquartile range, 43–63 years). In the entire study population, there were 449 (16.4%) patients with lymph node status N0, 568 (20.7%) patients with N1, 1234 (45.0%) patients with N2, and 490 (17.9%) patients with N3.

Furthermore, we explored the independent prognostic factors of OS and CSS in stage III–IVB NPC. The results of the univariate Cox analysis were shown in Table S2. The multivariate analysis was applied for factors with *p*‐values less than 0.05 in univariate analysis, in which stage variable was excluded considering the overlapping effects of stage variable, T stage variable, and N stage variable. As shown in Table S3, age at diagnosis, sex, race, marital status, grade, histology, T stage, N stage, radiotherapy, and chemotherapy were the independent prognostic indicators of OS. Additionally, the surgery to primary site variable was an independent prognostic factor for CSS.

### 3.2 Differences between patients receiving radiotherapy accompanied with or without chemotherapy before and after PSM

The results in Table S3 above indicated that patients with stage III–IVB NPC receiving radiotherapy and chemotherapy had benefits in terms of OS and CSS. Considering the latest version of the CSCO and ASCO guidelines recommending that all NPC patients require radiotherapy, we further compared the patients who received radiotherapy alone and those who received radiotherapy combined with chemotherapy to observe the clinicopathological difference between the two treatment groups.

**FIGURE 2:**
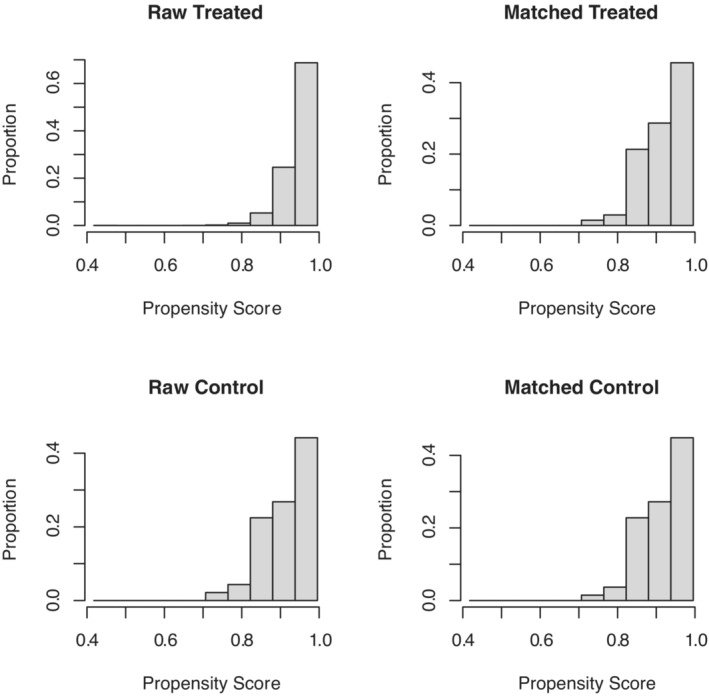
Distribution of propensity scores between the radiotherapy alone group and the chemoradiotherapy group before and after PSM

**FIGURE 3:**
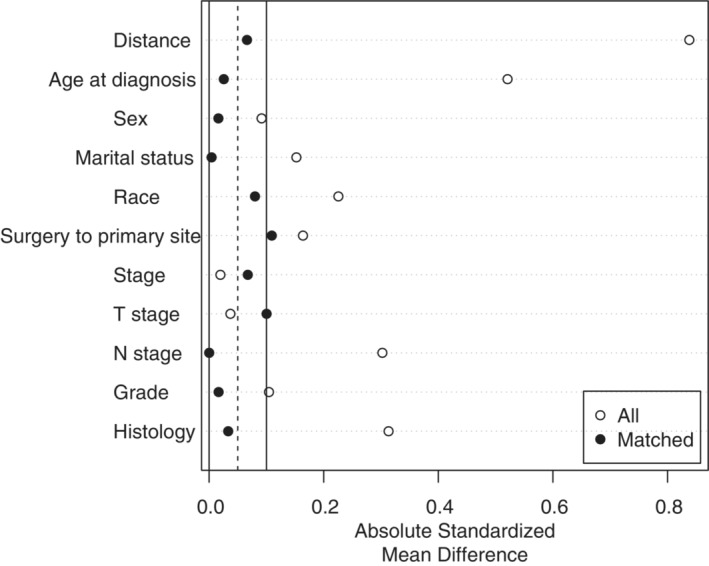
The standard deviation of each covariate between the two groups

As shown in Table 1, a total of 2463 patients who received radiotherapy were selected, including 138 patients who received radiotherapy alone and 2325 patients who received radiotherapy and chemotherapy. Between the two groups, we found that patients with younger age, higher pathological grade, and positive lymph nodes were more likely to receive radiotherapy combined with chemotherapy. The imbalance between the two treatment groups was significant, which inevitably led to selection bias. Therefore, we adopted the PSM method to match the radiotherapy alone group and the chemoradiotherapy group at a ratio of 1:1 to reduce the imbalance between covariates. During the calculation of the propensity score, we applied a logistic regression model with the therapy status as the dependent variable, and the nearest‐neighbor matching was used for all confounding factors (age at diagnosis, sex, race, marital status, grade, histology, stage, T stage, N stage, surgery to primary site). After PSM, the distribution of the propensity scores between the radiotherapy alone group and the chemoradiotherapy group became similar (Figure 2). Additionally, the difference in clinicopathological characteristics between the matched 136 pairs was significantly decreased and resulted in well balance across all covariates (Table S4 and Figure 3).

**TABLE 1:** Differences of clinicopathological variables between the patients with radiotherapy or chemoradiotherapy before PSM (*N* = 2463)

Characteristics	Total (*n* = 2463)	Radiotherapy (*n* = 138)	Chemoradiotherapy (*n* = 2325)	*p* value
Age at diagnosis				**<0.0001**
Mean ± SD	51.4 ± 15.3	58.8 ± 16.4	50.9 ± 15.2	
Sex				0.297
Male	1756 (71.3%)	93 (67.4%)	1663 (71.5%)	
Female	707 (28.7%)	45 (32.6%)	662 (28.5%)	
Race				**0.006**
White	1063 (43.2%)	73 (52.9%)	990 (42.6%)	
Black	331 (13.4%)	24 (17.4%)	307 (13.2%)	
Other^a^	1051 (42.7%)	40 (29.0%)	1011 (43.5%)	
Unknown	18 (0.7%)	1 (0.7%)	17 (0.7%)	
Marital status				0.181
Married	1399 (56.8%)	70 (50.7%)	1329 (57.2%)	
Unmarried	948 (38.5%)	58 (42.0%)	890 (38.3%)	
Unknown	116 (4.7%)	10 (7.2%)	106 (4.6%)	
Grade				**0.003**
I	32 (1.3%)	5 (3.6%)	27 (1.2%)	
II	197 (8.0%)	19 (13.8%)	178 (7.7%)	
III	775 (31.5%)	47 (34.1%)	728 (31.3%)	
IV	756 (30.7%)	29 (21.0%)	727 (31.3%)	
Unknown	703 (28.5%)	38 (27.5%)	665 (28.6%)	
Histology				**<0.0001**
KSCC	826 (33.5%)	72 (52.2%)	754 (32.4%)	
DNKSCC	678 (27.5%)	26 (18.8%)	652 (28.0%)	
UNKSCC	503 (20.4%)	20 (14.5%)	483 (20.8%)	
Other	456 (18.5%)	20 (14.5%)	436 (18.8%)	
Stage				0.899
III	1265 (51.4%)	71 (51.4%)	1194 (51.4%)	
IVA	755 (30.7%)	44 (31.9%)	711 (30.6%)	
IVB	443 (18.0%)	23 (16.7%)	420 (18.1%)	
T stage				0.927
T1	464 (18.8%)	28 (20.3%)	436 (18.8%)	
T2	425 (17.3%)	25 (18.1%)	400 (17.2%)	
T3	751 (30.5%)	39 (28.3%)	712 (30.6%)	
T4	823 (33.4%)	46 (33.3%)	777 (33.4%)	
N stage				**<0.0001**
N0	370 (15.0%)	39 (28.3%)	331 (14.2%)	
N1	497 (20.2%)	26 (18.8%)	471 (20.3%)	
N2	1153 (46.8%)	50 (36.2%)	1103 (47.4%)	
N3	443 (18.0%)	23 (16.7%)	420 (18.1%)	
Surgery to primary site				0.084
No	2242 (91.0%)	118 (85.5%)	2124 (91.4%)	
Yes	220 (8.9%)	20 (14.5%)	200 (8.6%)	
Unknown	1 (0%)	0 (0%)	1 (0%)	

*Note*: The bold values just mean statistically significant (*p* < 0.05).

Abbreviation: Other^a^, American Indian, Alaska Native, Asian, Pacific Islander.

### 3.3 Chemoradiotherapy effect for stage III–IVB NPC before and after PSM

**FIGURE 4:**
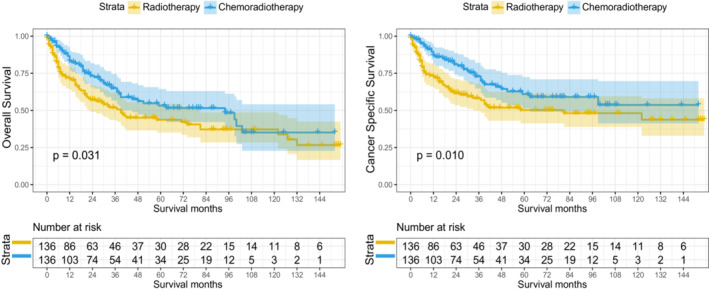
Kaplan–Meier curves of OS and CSS in stage III–IVB NPC after PSM

Multivariate Cox regression analysis was applied to all patients receiving radiotherapy before PSM (Table S5). We found that age at diagnosis, sex, marital status, grade, T stage, N stage, and therapy were independent prognostic factors for all patients receiving radiotherapy before PSM. In terms of therapeutic efficacy, we found that patients who received chemoradiotherapy had higher OS (*p* = 0.025) and CSS (*p* = 0.032) than those who received radiotherapy alone. Furthermore, Kaplan–Meier analyses were performed on the matched population after PSM (Figure 4). Compared with patients receiving radiotherapy alone, patients receiving chemoradiotherapy had significantly improved OS (*p* = 0.031) and CSS (*p* = 0.010).

### 3.4 Chemoradiotherapy showed survival benefits for node‐positive stage III–IVB NPC

**FIGURE 5:**
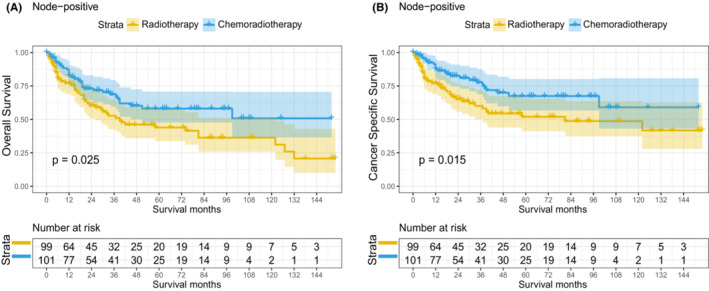
Kaplan–Meier curves of OS and CSS in node‐positive stage III–IVBNPC

In matched population, 226 patients were diagnosed with stage III–IVA and 46 patients were stage IVB (T1–4N3M0). Among the stage III–IVA patients, 72 patients were diagnosed with stage N0 (T3–4N0M0), 56 patients were stage N1 (T1–4N1M0), and 98 patients were stage N2 (T1–4N2M0). We further explored the survival benefit of two treatment methods in stage III–IVB patients stratified by lymph node status. Multivariate cox analysis was shown in Table 2 and Table S6, elderly patients (HR 1.063, 95% CI 1.033–1.093), higher T stage (HR 3.933, 95% CI 1.530–10.110), and chemoradiotherapy (HR 0.488, 95% CI 0.250–0.952) were independent prognostic indicators of OS for node‐positive stage III–IVB NPC, and the above results were similar to those of CSS. In the Kaplan–Meier curve, the 5‐year OS rates (58.08% vs. 43.95%; *p* = 0.025; Figure 5A) and 5‐year CSS rates (67.42% vs. 51.95%; *p* = 0.015; Figure 5B) in the chemoradiotherapy group were significantly higher than those in the radiotherapy alone group.

**TABLE 2:** Multivariate cox analysis of OS and CSS in all Node‐positive stage III–IVB NPC with radiotherapy after PSM (*N* = 200)

Variables	OS		CSS	
	HR (95% CI)	*p* value	HR (95% CI)	*p* value
Age at diagnosis	1.063 (1.033–1.093)	**<0.0001**	1.042 (1.011–1.074)	**0.008**
T stage		**0.004**		**0.006**
T1	Reference		Reference	
T2	0.576 (0.216–1.540)	0.272	0.587 (0.173–1.994)	0.393
T3	3.933 (1.530–10.110)	0.004	5.027 (1.732–14.585)	0.003
T4	2.769 (0.846–9.056)	0.092	3.294 (0.854–12.696)	0.083
Therapy		**0.035**		**0.014**
Radiotherapy	Reference		Reference	
Chemoradiotherapy	0.488 (0.250–0.952)	0.035	0.366 (0.165–0.814)	0.014

*Note*: The bold values just mean statistically significant (*p* < 0.05).

### 3.5 Chemoradiotherapy showed no survival benefit for node‐negative stage III–IVA NPC

**FIGURE 6:**
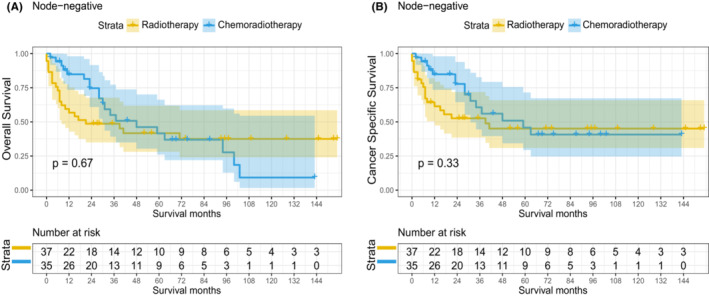
Kaplan–Meier curves of OS and CSS in node‐negative stage III–IVA NPC

After multivariate cox analysis (Table 3 and Table S7), we identify elderly patients and higher T stage were independent prognostic risk factors of OS for patient node‐negative stage III–IVA NPC. Interestingly, chemoradiotherapy showed no OS (*p* = 0.326) or CSS (*p* = 0.155) benefit compared with radiotherapy alone. Kaplan–Meier curve illustrated there was no difference in 5‐year OS rates between the chemoradiotherapy group and radiotherapy alone group (41.55% vs. 44.08%; *p* = 0.67; Figure 6A). Additionally, chemoradiotherapy also did not improve 5‐year CSS rates compared to radiotherapy alone (45.82% vs. 47.67%; *p* = 0.33; Figure 6B).

**TABLE 3:** Multivariate cox analysis of OS and CSS in all node‐negative stage III–IVA NPC with radiotherapy after PSM (*N* = 72)

Variables	OS		CSS	
	HR (95% CI)	*p* value	HR (95% CI)	*p* value
Age at diagnosis	1.057 (1.016–1.099)	**0.006**	1.035 (0.995–1.076)	0.086
T stage		**0.028**		0.055
T3	Reference		Reference	
T4	3.049 (1.130–8.233)	0.028	2.848 (0.977–8.299)	0.055

*Note*: The bold values just mean statistically significant (*p* < 0.05).

Furthermore, we included a total of 449 lymph node‐negative stage III–IVA (T3–4N0M0) NPC patients from the 2741 total study population to explore their prognostic factors. The results of the univariate analysis were elaborated in Table S8, in which statistically significant variables were further included in multivariate analysis. Ultimately, the results in Table 4 explicated that age at diagnosis, marital status, grade, and radiotherapy variables were independent indicators of OS and CSS. Patients who received radiotherapy had better OS rates (HR 0.293, 95% CI 0.203–0.424) and CSS rates (HR 0.252, 95% CI 0.171–0.371) than those who did not received radiotherapy. However, the chemotherapy variable did not show significant statistical significance in multivariate analysis.

**TABLE 4:** Multivariate cox analysis of OS and CSS in all stage T3–4N0M0 NPC (*N* = 449)

Variables	OS		CSS	
	HR (95% CI)	*p* value	HR (95% CI)	*p* value
Age at diagnosis	1.034 (1.021–1.047)	**<0.0001**	1.029 (1.016–1.043)	**<0.0001**
Marital status		**0.005**		**0.009**
Married	Reference		Reference	
Unmarried	1.543 (1.136–2.095)	0.005	1.554 (1.114–2.168)	0.009
Grade		**<0.0001**		**<0.0001**
I	Reference		Reference	
II	1.621 (0.793–3.311)	0.185	1.682 (0.787–3.591)	0.179
III	0.897 (0.445–1.807)	0.761	0.863 (0.409–1.821)	0.699
IV	0.528 (0.246–1.137)	0.103	0.563 (0.249–1.273)	0.168
Radiotherapy		**<0.0001**		**<0.0001**
No	Reference		Reference	
Yes	0.293 (0.203–0.424)	<0.0001	0.252 (0.171–0.371)	<0.0001

*Note*: The bold values just mean statistically significant (*p* < 0.05).

## 4 DISCUSSION

Our research revealed that based on radiotherapy alone, the addition of chemotherapy significantly improved the OS and CSS rates of patients with advanced NPC, especially for those with positive lymph nodes, while those with negative lymph nodes may not benefit from additional chemotherapy.

CCRT is considered to be the main treatment for locoregionally advanced NPC. The evidence sources mainly include several randomized clinical studies such as intergroup 0099^22^ and a meta‐analysis of 4806 patients with NPC.^23^ The results showed that CCRT had significant benefits over radiotherapy alone in OS and PFS. However, the scope of the population included in the above study covered patients with stages ranging from stage II to IVA, and the real beneficiaries needed to be further discussed. As mentioned earlier, some studies have found that CCRT combined with IC or AC can significantly benefit patients with advanced NPC,^6^, ^7^, ^24^, ^25^ which is consistent with our results that chemotherapy would indeed improve the OS and CSS rated of stage III–IVA (8th edition of AJCC staging) NPC. However, when we further stratify the lymph node status to explore more, we found that patients with node‐positive disease can benefit from additional chemotherapy, while those with node‐negative disease may not benefit from it. We speculate that this may be due to the serious side effects of patients with negative lymph nodes caused by radiotherapy combined with chemotherapy, which affects the efficacy and survival rates of patients.

Our results conflict with the latest CSCO and ASCO guidelines in 2021. Theoretically, radiotherapy combined with chemotherapy has many advantages: (a) Chemotherapy enables to eliminate or inhibit the possible micrometastasis and reduce the rates of distant metastasis; (b) Chemotherapy can also reduce the tumor burden, improve the oxygenation state in the center of the tumor and improve the sensitivity of radiotherapy.^26^, ^27^ But some researchers still come to different conclusions. Zhang et al.^28^ retrospectively analyzed the efficacy of IC combined with radiotherapy alone and IC combined with CCRT in 120 cases of locally advanced NPC, and their results showed that there was no significant difference in local control rates, distant metastasis rates, and OS rates between the two groups. The Hong Kong NPC9901/9902 series of studies compared the effects of CCRT and radiotherapy alone for locally advanced NPC. The 3‐year^19^ and 5‐year^29^ follow‐up results of the NPC9901 study both showed that the local control rates of the CCRT group were significantly higher than that of the radiotherapy alone group, while there was no significant difference in the distant metastasis rates and OS rates between the two groups. The 3‐year follow‐up results of the NPC9902 study also did not show the advantages of CCRT over radiotherapy alone.^30^ In 2017, it was reported that the 10‐year follow‐up results of the NPC9901 study^31^ were consistent with the results of the previous combined analysis^32^: the CCRT group had a significant benefit over the OS rates of radiotherapy alone group for patients with stage III (T1–3N2M0), but no similar benefit was seen in stage IV patients. A recent SEER‐based study also showed that stage T3N0M0 NPC did not benefit from chemotherapy,^33^ which is similar to our results. In addition to the lack of benefit from chemotherapy as described in the above studies, there are even several studies suggesting that the addition of chemotherapy may lead to an increase in toxic and side effects.^34^, ^35^

Based on the above, patients who may be exempted from concurrent chemotherapy should be screened out, and patients with negative lymph nodes in the locoregionally advanced low‐risk group should be explored from the perspective of clinical staging. Our study stratified the benefits of chemotherapy according to the lymph node status of NPC. Our study still has inevitable defects: First, it is a pity that the SEER database does not collect specific information of the treatment, such as the strategy and sequence of chemotherapy, the technology, and dose of radiotherapy, as well as the status of Epstein–Barr virus DNA^36^ and disease recurrence, which may limit the value of this research; Second, selection bias and competitive risk are inevitable due to retrospective analysis, but we have tried to use statistical methods such as PSM analysis, multivariate analyses and subgroup analyses to minimize the influence of confounding factors; Third, some researchers still have some concerns about the integrity and potential deviation of chemotherapy data provided by SEER database.^37^ Nevertheless, we are confident that the SEER database will accurately collect radiation and chemotherapy data for each individual, which is sufficient to support our current analysis.

To our knowledge, this is the first study to use a large sample obtained from the SEER database to expound the efficacy of chemotherapy for patients with stage III–IVA NPC based on lymph node status. Our results showed that for patients with negative lymph nodes, radiotherapy combined chemotherapy did not show a significant advantage over radiotherapy alone. Therefore, in the future, it is necessary to combine clinical staging, prognostic risk stratification, and prognostic‐related biomarkers to stratify patients and implement precise policies to further improve the efficacy and reduce treatment‐related toxic and side effects. The results of this study urgently require prospective clinical trials to clarify appropriate treatments for this subgroup.

## AUTHOR CONTRIBUTIONS

**Weiling Qu:** Conceptualization (lead); data curation (lead); formal analysis (equal); funding acquisition (equal); investigation (lead); methodology (lead); project administration (equal); resources (equal); software (equal); supervision (equal); validation (equal); visualization (equal); writing – original draft (lead); writing – review and editing (lead). **Xuan Wang:** Data curation (supporting); formal analysis (equal); investigation (equal); methodology (supporting); resources (supporting); software (supporting); supervision (equal); validation (equal); visualization (equal). **Qiao Qiao:** Conceptualization (supporting); data curation (equal); formal analysis (equal); funding acquisition (lead); investigation (equal); methodology (equal); project administration (supporting); resources (supporting); software (supporting); supervision (equal); validation (supporting); visualization (supporting); writing – original draft (supporting); writing – review and editing (supporting). **Yanli Wang:** Conceptualization (lead); data curation (equal); formal analysis (equal); funding acquisition (lead); investigation (equal); methodology (equal); project administration (equal); resources (equal); software (supporting); supervision (equal); validation (equal); visualization (supporting); writing – original draft (equal); writing – review and editing (equal).

## FUNDING INFORMATION

This research was funded by the Beijing Xisike Clinical Oncology Research Foundation (grant no. Y‐HR2019‐0326) and the Shenyang Science and Technology Bureau (grant no. RC210153 and 21‐172‐9‐14).

## CONFLICT OF INTEREST

The authors have no conflict of interest to declare.

## ETHICS APPROVAL STATEMENT

Not applicable. This is a retrospective study. The data of this paper was downloaded and obtained from the National Cancer Institute SEER program. We consulted extensively with the relevant responsible persons who determined that our study did not need ethical approval, as anonymous data were used.

## Supporting information


Table S1

Table S2

Table S3

Table S4

Table S5

Table S6

Table S7

Table S8

## Data Availability

Publicly available datasets were analyzed in this study. This data can be found here: https://seer.cancer.gov/data/.
