# Supplementary material for: Chemotherapy for locoregionally advanced nasopharyngeal carcinoma: Who really needs it
Source: Cancer Med. 2022 Dec 9;12(6):6994–7004. doi: 10.1002/cam4.5497 (PMC10067101; doi:10.1002/cam4.5497)
Supplement: Supplementary file 1 — Table S1 [file CAM4-12-6994-s002.docx]

**Table S1: Baseline clinical characteristics (N=2741)**

| **Characteristics** | **Level** | **Number (%)** |
| --- | --- | --- |
| **Age at diagnosis (years)** | Mean ± SD | 52.0 ± 15.4 |
|  | Median (range) | 53 (43-63) |
| **Sex** | Male | 1965 (71.7) |
|  | Female | 776 (28.3) |
| **Race** | White | 1198 (43.7) |
|  | Black | 375 (13.7) |
|  | Other^a^ | 1148 (41.9) |
|  | Unknown | 20 (0.7) |
| **Marital status** | Married | 1521 (55.5) |
|  | Unmarried | 1087 (39.7) |
|  | Unknown | 133 (4.9) |
| **Grade** | I | 42 (1.5) |
|  | II | 246 (9.0) |
|  | III | 855 (31.2) |
|  | IV | 809 (29.5) |
|  | Unknown | 789 (28.8) |
| **Histology** | KSCC | 968 (35.3) |
|  | DNKSCC | 727 (26.5) |
|  | UNKSCC | 536 (19.6) |
|  | Other | 510 (18.6) |
| **Stage** | III | 1388 (50.6) |
|  | IVA | 863 (31.5) |
|  | IVB | 490 (17.9) |
| **T stage** | T1 | 495 (18.1) |
|  | T2 | 451 (16.5) |
|  | T3 | 846 (30.9) |
|  | T4 | 949 (34.6) |
| **N stage** | N0 | 449 (16.4) |
|  | N1 | 568 (20.7) |
|  | N2 | 1234 (45.0) |
|  | N3 | 490 (17.9) |
| **Surgery to primary site** | No | 2499 (91.2) |
|  | Yes | 240 (8.8) |
|  | Unknown | 2 (0.1) |
| **Radiotherapy** | No | 278 (10.1) |
|  | Yes | 2463 (89.9) |
| **Chemotherapy** | No | 317 (11.6) |
|  | Yes | 2424 (88.4) |

**Abbreviations:** Other^a^, American Indian, Alaska Native, Asian, Pacific Islander.
