# Supplementary material for: Chemotherapy for locoregionally advanced nasopharyngeal carcinoma: Who really needs it
Source: Cancer Med. 2022 Dec 9;12(6):6994–7004. doi: 10.1002/cam4.5497 (PMC10067101; doi:10.1002/cam4.5497)
Supplement: Supplementary file 2 — Table S2 [file CAM4-12-6994-s005.docx]

**Table S2: Univariate cox analysis of OS and CSS in all stage III-IVB NPC (N=2741)**

| **Variables** | **OS** | | **CSS** | |
| --- | --- | --- | --- | --- |
|  | **HR (95% CI)** | ***P* value** | **HR (95% CI)** | ***P* value** |
| **Age at diagnosis** | 1.041 (1.036-1.046) | **<0.0001** | 1.035 (1.030-1.040) | **<0.0001** |
| **Sex** |  | **0.032** |  | 0.066 |
| Male | Reference |  | Reference |  |
| Female | 0.858 (0.747-0.987) | 0.032 | 0.866 (0.743-1.009) | 0.066 |
| **Race** |  | **<0.0001** |  | **<0.0001** |
| White | Reference |  | Reference |  |
| Black | 0.892 (0.745-1.067) | 0.210 | 0.903 (0.741-1.100) | 0.311 |
| Other^a^ | 0.586 (0.512-0.671) | <0.0001 | 0.597 (0.515-0.693) | <0.0001 |
| **Marital status** |  | **<0.0001** |  | **<0.0001** |
| Married | Reference |  | Reference |  |
| Unmarried | 1.286 (1.135-1.458) | <0.0001 | 1.353 (1.179-1.553) | <0.0001 |
| **Grade** |  | **<0.0001** |  | **<0.0001** |
| I | Reference |  | Reference |  |
| II | 1.183 (0.769-1.818) | 0.445 | 1.240 (0.775-1.984) | 0.369 |
| III | 0.537 (0.355-0.813) | 0.003 | 0.523 (0.332-0.824) | 0.005 |
| IV | 0.343 (0.226-0.523) | <0.0001 | 0.357 (0.226-0.567) | <0.0001 |
| **Histology** |  | **<0.0001** |  | **<0.0001** |
| KSCC | Reference |  | Reference |  |
| DNKSCC | 0.551 (0.471-0.645) | <0.0001 | 0.552 (0.465-0.656) | <0.0001 |
| UNKSCC | 0.408 (0.340-0.489) | <0.0001 | 0.406 (0.332-0.496) | <0.0001 |
| Other | 0.516 (0.432-0.616) | <0.0001 | 0.491 (0.403-0.598) | <0.0001 |
| **Stage** |  | **<0.0001** |  | **<0.0001** |
| III | Reference |  | Reference |  |
| IVA | 1.607 (1.403-1.842) | <0.0001 | 1.600 (1.377-1.860) | <0.0001 |
| IVB | 1.351 (1.143-1.597) | <0.0001 | 1.373 (1.143-1.648) | 0.001 |
| **T stage** |  | **<0.0001** |  | **<0.0001** |
| T1 | Reference |  | Reference |  |
| T2 | 1.251 (0.986-1.588) | 0.066 | 1.261 (0.970-1.639) | 0.083 |
| T3 | 1.766 (1.439-2.167) | <0.0001 | 1.748 (1.395-2.190) | <0.0001 |
| T4 | 2.177 (1.782-2.658) | <0.0001 | 2.148 (1.724-2.677) | <0.0001 |
| **N stage** |  | **<0.0001** |  | **<0.0001** |
| N0 | Reference |  | Reference |  |
| N1 | 0.637 (0.529-0.767) | <0.0001 | 0.610 (0.497-0.749) | <0.0001 |
| N2 | 0.510 (0.434-0.600) | <0.0001 | 0.508 (0.425-0.606) | <0.0001 |
| N3 | 0.704 (0.582-0.850) | <0.0001 | 0.706 (0.574-0.869) | 0.001 |
| **Surgery to primary site** |  | 0.092 |  | **0.024** |
| No | Reference |  |  |  |
| Yes | 0.826 (0.660-1.032) | 0.092 | 0.745 (0.576-0.962) | 0.024 |
| **Radiotherapy** |  | **<0.0001** |  | **<0.0001** |
| No | Reference |  | Reference |  |
| Yes | 0.261 (0.222-0.305) | <0.0001 | 0.245 (0.207-0.291) | <0.0001 |
| **Chemotherapy** |  | **<0.0001** |  | **<0.0001** |
| No | Reference |  | Reference |  |
| Yes | 0.330 (0.283-0.384) | <0.0001 | 0.324 (0.274-0.383) | <0.0001 |

**Abbreviations:** Other^a^, American Indian, Alaska Native, Asian, Pacific Islander.
