# Supplementary material for: Chemotherapy for locoregionally advanced nasopharyngeal carcinoma: Who really needs it
Source: Cancer Med. 2022 Dec 9;12(6):6994–7004. doi: 10.1002/cam4.5497 (PMC10067101; doi:10.1002/cam4.5497)
Supplement: Supplementary file 3 — Table S3 [file CAM4-12-6994-s008.docx]

**Table S3: Multivariate cox analysis of OS and CSS in all stage III-IVB NPC (N=2741)**

| **Variables** | **OS** | | **CSS** | |
| --- | --- | --- | --- | --- |
|  | **HR (95% CI)** | ***P* value** | **HR (95% CI)** | ***P* value** |
| **Age at diagnosis** | 1.038 (1.032-1.044) | **<0.0001** | 1.031 (1.025-1.037) | **<0.0001** |
| **Sex** |  | **0.044** |  | **-** |
| Male | Reference |  | - | **-** |
| Female | 0.836 (0.702-0.995) | 0.044 | - | **-** |
| **Race** |  | **0.034** |  | 0.056 |
| White | Reference |  | Reference |  |
| Black | 1.094 (0.875-1.368) | 0.432 | 1.025 (0.801-1.312) | 0.844 |
| Other^a^ | 0.820 (0.685-0.982) | 0.031 | 0.798 (0.656-0.973) | 0.025 |
| **Marital status** |  | **<0.0001** |  | **<0.0001** |
| Married | Reference |  | Reference |  |
| Unmarried | 1.492 (1.274-1.748) | <0.0001 | 1.429 (1.207-1.691) | <0.0001 |
| **Grade** |  | **0.0004** |  | **<0.0001** |
| I | Reference |  | Reference |  |
| II | 1.184 (0.764-1.833) | 0.450 | 1.274 (0.790-2.056) | 0.321 |
| III | 0.753 (0.493-1.149) | 0.188 | 0.757 (0.477-1.203) | 0.239 |
| IV | 0.790 (0.497-1.255) | 0.319 | 0.805 (0.486-1.335) | 0.401 |
| **Histology** |  | **0.046** |  | 0.135 |
| KSCC | Reference |  | Reference |  |
| DNKSCC | 0.791 (0.641-0.976) | 0.029 | 0.844 (0.672-1.061) | 0.146 |
| UNKSCC | 0.678 (0.503-0.912) | 0.010 | 0.682 (0.493-0.943) | 0.021 |
| Other | 0.851 (0.659-1.100) | 0.218 | 0.853 (0.642-1.133) | 0.271 |
| **T stage** |  | **<0.0001** |  | **<0.0001** |
| T1 | Reference |  | Reference |  |
| T2 | 1.467 (1.100-1.956) | 0.009 | 1.502 (1.094-2.063) | 0.012 |
| T3 | 1.843 (1.407-2.413) | <0.0001 | 1.833 (1.363-2.463) | <0.0001 |
| T4 | 2.221 (1.697-2.907) | <0.0001 | 2.179 (1.624-2.925) | <0.0001 |
| **N stage** |  | **0.007** |  | **0.014** |
| N0 | Reference |  | Reference |  |
| N1 | 0.807 (0.642-1.013) | 0.065 | 0.789 (0.614-1.013) | 0.063 |
| N2 | 0.962 (0.774-1.195) | 0.724 | 0.939 (0.740-1.191) | 0.605 |
| N3 | 1.262 (0.977-1.629) | 0.074 | 1.237 (0.937-1.633) | 0.134 |
| **Surgery to primary site** |  | - |  | **0.018** |
| No | - | - | Reference |  |
| Yes | - | - | 0.693 (0.512-0.939) | 0.018 |
| **Radiotherapy** |  | **<0.0001** |  | **<0.0001** |
| No | Reference |  | Reference |  |
| Yes | 0.346 (0.272-0.439) | <0.0001 | 0.312 (0.241-0.404) | <0.0001 |
| **Chemotherapy** |  | **0.014** |  | **0.011** |
| No | Reference |  | Reference |  |
| Yes | 0.743 (0.586-0.942) | 0.014 | 0.709 (0.545-0.923) | 0.011 |

**Abbreviations:** Other^a^, American Indian, Alaska Native, Asian, Pacific Islander.
