# Supplementary material for: Chemotherapy for locoregionally advanced nasopharyngeal carcinoma: Who really needs it
Source: Cancer Med. 2022 Dec 9;12(6):6994–7004. doi: 10.1002/cam4.5497 (PMC10067101; doi:10.1002/cam4.5497)
Supplement: Supplementary file 4 — Table S4 [file CAM4-12-6994-s007.docx]

**Table S4: Differences of clinicopathological variables between the patients with radiotherapy or chemoradiotherapy after PSM (N=272)**

| **Characteristics** | **Total (n=272)** | **Radiotherapy (n=136)** | **Chemoradiotherapy (n=136)** | ***P* value** |
| --- | --- | --- | --- | --- |
| **Age at diagnosis** |  |  |  | 0.833 |
| Mean ± SD | 58.2 ± 15.2 | 58.4 ± 16.2 | 58.0 ± 14.2 |  |
| **Sex** |  |  |  | 0.897 |
| Male | 185 (68.0%) | 93 (68.4%) | 92 (67.6%) |  |
| Female | 87 (32.0%) | 43 (31.6%) | 44 (32.4%) |  |
| **Race** |  |  |  | 0.381 |
| White | 142 (52.2%) | 72 (52.9%) | 70 (51.5%) |  |
| Black | 40 (14.7%) | 24 (17.6%) | 16 (11.8%) |  |
| Other^a^ | 88 (32.4%) | 39 (28.7%) | 49 (36.0%) |  |
| Unknown | 2 (0.7%) | 1 (0.7%) | 1 (0.7%) |  |
| **Marital status** |  |  |  | 0.684 |
| Married | 146 (53.7%) | 70 (51.5%) | 76 (55.9%) |  |
| Unmarried | 105 (38.6%) | 56 (41.2%) | 49 (36.0%) |  |
| Unknown | 21 (7.7%) | 10 (7.4%) | 11 (8.1%) |  |
| **Grade** |  |  |  | 0.517 |
| I | 6 (2.2%) | 5 (3.7%) | 1 (0.7%) |  |
| II | 33 (12.1%) | 18 (13.2%) | 15 (11.0%) |  |
| III | 99 (36.4%) | 46 (33.8%) | 53 (39.0%) |  |
| IV | 59 (21.7%) | 29 (21.3%) | 30 (22.1%) |  |
| Unknown | 75 (27.6%) | 38 (27.9%) | 37 (27.2%) |  |
| **Histology** |  |  |  | 0.717 |
| KSCC | 134 (49.3%) | 70 (51.5%) | 64 (47.1%) |  |
| DNKSCC | 56 (20.6%) | 26 (19.1%) | 30 (22.1%) |  |
| UNKSCC | 45 (16.5%) | 20 (14.7%) | 25 (18.4%) |  |
| Other | 37 (13.6%) | 20 (14.7%) | 17 (12.5%) |  |
| **Stage** |  |  |  | 0.641 |
| III | 131 (48.2%) | 69 (50.7%) | 62 (45.6%) |  |
| IVA | 95 (34.9%) | 44 (32.4%) | 51 (37.5%) |  |
| IVB | 46 (16.9%) | 23 (16.9%) | 23 (16.9%) |  |
| **T stage** |  |  |  | 0.854 |
| T1 | 52 (19.1%) | 28 (20.6%) | 24 (17.6%) |  |
| T2 | 47 (17.3%) | 25 (18.4%) | 22 (16.2%) |  |
| T3 | 77 (28.3%) | 37 (27.2%) | 40 (29.4%) |  |
| T4 | 96 (35.3%) | 46 (33.8%) | 50 (36.8%) |  |
| **N stage** |  |  |  | 0.944 |
| N0 | 72 (26.5%) | 37 (27.2%) | 35 (25.7%) |  |
| N1 | 56 (20.6%) | 26 (19.1%) | 30 (22.1%) |  |
| N2 | 98 (36.0%) | 50 (36.8%) | 48 (35.3%) |  |
| N3 | 46 (16.9%) | 23 (16.9%) | 23 (16.9%) |  |
| **Surgery to primary site** |  |  |  | 0.365 |
| No | 237 (87.1%) | 116 (85.3%) | 121 (89.0%) |  |
| Yes | 35 (12.9%) | 20 (14.7%) | 15 (11.0%) |  |

**Abbreviations:** Other^a^, American Indian, Alaska Native, Asian, Pacific Islander.
