# Supplementary material for: Chemotherapy for locoregionally advanced nasopharyngeal carcinoma: Who really needs it
Source: Cancer Med. 2022 Dec 9;12(6):6994–7004. doi: 10.1002/cam4.5497 (PMC10067101; doi:10.1002/cam4.5497)
Supplement: Supplementary file 5 — Table S5 [file CAM4-12-6994-s003.docx]

**Table S5: Multivariate cox analysis of OS and CSS in all stage III-IVB NPC with radiotherapy before PSM (N=2463)**

| **Variables** | **OS** | | **CSS** | |
| --- | --- | --- | --- | --- |
|  | **HR (95% CI)** | ***P* value** | **HR (95% CI)** | ***P* value** |
| **Age at diagnosis** | 1.037 (1.031-1.044) | **<0.0001** | 1.029 (1.023-1.036) | **<0.0001** |
| **Sex** |  | **0.037** |  | 0.120 |
| Male | Reference |  | Reference |  |
| Female | 0.818 (0.677-0.988) | 0.037 | 0.849 (0.690-1.044) | 0.120 |
| **Race** |  | 0.194 |  | 0.329 |
| White | Reference |  | Reference |  |
| Black | 1.103 (0.853-1.426) | 0.457 | 1.049 (0.788-1.397) | 0.743 |
| Other^a^ | 0.871 (0.714-1.063) | 0.174 | 0.865 (0.695-1.077) | 0.194 |
| **Marital status** |  | **<0.0001** |  | **0.005** |
| Married | Reference |  | Reference |  |
| Unmarried | 1.386 (1.162-1.653) | <0.0001 | 1.322 (1.089-1.606) | 0.005 |
| **Grade** |  | **0.010** |  | **0.006** |
| I | Reference |  | Reference |  |
| II | 1.008 (0.599-1.697) | 0.975 | 1.154 (0.639-2.083) | 0.635 |
| III | 0.686 (0.418-1.124) | 0.135 | 0.725 (0.413-1.275) | 0.265 |
| IV | 0.654 (0.384-1.114) | 0.118 | 0.701 (0.383-1.280) | 0.247 |
| **Histology** |  | 0.434 |  | 0.533 |
| KSCC | Reference |  | Reference |  |
| DNKSCC | 0.908 (0.725-1.138) | 0.402 | 1.001 (0.782-1.281) | 0.993 |
| UNKSCC | 0.785 (0.572-1.076) | 0.133 | 0.806 (0.569-1.141) | 0.225 |
| Other | 0.973 (0.734-1.290) | 0.849 | 1.002 (0.731-1.372) | 0.992 |
| **T stage** |  | **<0.0001** |  | **0.001** |
| T1 | Reference |  | Reference |  |
| T2 | 1.438 (1.059-1.951) | 0.020 | 1.409 (1.006-1.973) | 0.046 |
| T3 | 1.847 (1.382-2.468) | <0.0001 | 1.824 (1.327-2.509) | <0.0001 |
| T4 | 2.286 (1.711-3.055) | <0.0001 | 2.225 (1.619-3.056) | <0.0001 |
| **N stage** |  | **<0.0001** |  | **<0.0001** |
| N0 | Reference |  | Reference |  |
| N1 | 0.730 (0.564-0.945) | 0.017 | 0.716 (0.537-0.955) | 0.023 |
| N2 | 0.974 (0.765-1.240) | 0.829 | 0.971 (0.743-1.269) | 0.827 |
| N3 | 1.490 (1.125-1.975) | 0.005 | 1.451 (1.063-1.980) | 0.019 |
| **Surgery to primary site** |  | 0.106 |  | **0.045** |
| No | Reference |  | Reference |  |
| Yes | 0.785 (0.585-1.053) | 0.106 | 0.710 (0.508-0.992) | 0.045 |
| **Therapy** |  | **0.025** |  | **0.032** |
| Radiotherapy | Reference |  | Reference |  |
| Chemoradiotherapy | 0.706 (0.521-0.958) | 0.025 | 0.689 (0.490-0.968) | 0.032 |

**Abbreviations:** Other^a^, American Indian, Alaska Native, Asian, Pacific Islander.
