# Supplementary material for: Chemotherapy for locoregionally advanced nasopharyngeal carcinoma: Who really needs it
Source: Cancer Med. 2022 Dec 9;12(6):6994–7004. doi: 10.1002/cam4.5497 (PMC10067101; doi:10.1002/cam4.5497)
Supplement: Supplementary file 6 — Table S6 [file CAM4-12-6994-s004.docx]

**Table S6: Multivariate cox analysis of OS and CSS in all Node-positive stage III-IVB NPC with radiotherapy after PSM (N=200)**

| **Variables** | **OS** | | **CSS** | |
| --- | --- | --- | --- | --- |
|  | **HR (95% CI)** | ***P* value** | **HR (95% CI)** | ***P* value** |
| **Sex** |  | 0.549 |  | 0.285 |
| Male | Reference |  | Reference |  |
| Female | 1.224 (0.632-2.369) | 0.549 | 1.524 (0.704-3.300) | 0.285 |
| **Race** |  | 0.086 |  | 0.199 |
| White | Reference |  | Reference |  |
| Black | 1.775 (0.739-4.266) | 0.199 | 1.661 (0.599-4.607) | 0.330 |
| Other^a^ | 0.553 (0.247-1.238) | 0.150 | 0.546 (0.211-1.412) | 0.212 |
| **Marital status** |  | 0.429 |  | 0.396 |
| Married | Reference |  | Reference |  |
| Unmarried | 0.740 (0.350-1.562) | 0.429 | 0.687 (0.288-1.636) | 0.396 |
| **Grade** |  | 0.843 |  | 0.452 |
| I | Reference |  | Reference |  |
| II | 2.431 (0.245-24.095) | 0.448 | - | 0.918 |
| III | 1.804 (0.196-16.644) | 0.603 | - | 0.924 |
| IV | 2.032 (0.182-22.660) | 0.564 | - | 0.923 |
| **Histology** |  | 0.935 |  | 0.918 |
| KSCC | Reference |  | Reference |  |
| DNKSCC | 0.743 (0.289-1.908) | 0.537 | 0.956 (0.322-2.835) | 0.935 |
| UNKSCC | 0.954 (0.257-3.541) | 0.944 | 0.985 (0.212-4.586) | 0.985 |
| Other | 0.954 (0.350-2.596) | 0.926 | 1.395 (0.466-4.178) | 0.552 |
| **N stage** |  | 0.205 |  | 0.206 |
| N1 | Reference |  | Reference |  |
| N2 | 2.386 (0.865-6.579) | 0.093 | 2.858 (0.887-9.212) | 0.079 |
| N3 | 2.195 (0.808-5.961) | 0.123 | 2.160 (0.698-6.682) | 0.181 |
| **Surgery to primary site** |  | 0.085 |  | 0.099 |
| No | Reference |  | Reference |  |
| Yes | 0.438 (0.171-1.122) | 0.085 | 0.409 (0.142-1.184) | 0.099 |

**Abbreviations:** Other^a^, American Indian, Alaska Native, Asian, Pacific Islande
