# Supplementary material for: Chemotherapy for locoregionally advanced nasopharyngeal carcinoma: Who really needs it
Source: Cancer Med. 2022 Dec 9;12(6):6994–7004. doi: 10.1002/cam4.5497 (PMC10067101; doi:10.1002/cam4.5497)
Supplement: Supplementary file 7 — Table S7 [file CAM4-12-6994-s001.docx]

**Table S7: Multivariate cox analysis of OS and CSS in all Node-negative stage III-IVA NPC with radiotherapy after PSM (N=72)**

| **Variables** | **OS** | | **CSS** | |
| --- | --- | --- | --- | --- |
|  | **HR (95% CI)** | ***P* value** | **HR (95% CI)** | ***P* value** |
| **Sex** |  | 0.214 |  | 0.257 |
| Male | Reference |  | Reference |  |
| Female | 0.556 (0.221-1.402) | 0.214 | 0.569 (0.215-1.508) | 0.257 |
| **Race** |  | 0.830 |  | 0.733 |
| White | Reference |  | Reference |  |
| Black | 0.944 (0.271-3.281) | 0.927 | 0.597 (0.152-2.351) | 0.461 |
| Other^a^ | 0.675 (0.190-2.398) | 0.543 | 0.710 (0.168-3.007) | 0.642 |
| **Marital status** |  | 0.994 |  | 0.459 |
| Married | Reference |  | Reference |  |
| Unmarried | 1.004 (0.367-2.750) | 0.994 | 1.510 (0.507-4.495) | 0.459 |
| **Grade** |  | 0.876 |  | 0.944 |
| I | Reference |  | Reference |  |
| II | 0.581 (0.126-2.668) | 0.485 | 1.261 (0.206-7.719) | 0.802 |
| III | 0.588 (0.125-2.779) | 0.503 | 0.843 (0.140-5.070) | 0.852 |
| IV | 0.791 (0.077-8.105) | 0.843 | 1.087 (0.089-13.335) | 0.948 |
| **Histology** |  | 0.542 |  | 0.769 |
| KSCC | Reference |  | Reference |  |
| DNKSCC | 0.412 (0.099-1.705) | 0.221 | 0.501 (0.104-2.407) | 0.388 |
| UNKSCC | 1.483 (0.206-10.693) | 0.696 | 1.376 (0.169-11.229) | 0.766 |
| Other | 0.938 (0.128-6.858) | 0.949 | 1.131 (0.153-8.340) | 0.904 |
| **Surgery to primary site** |  | 0.634 |  | 0.794 |
| No | Reference |  | Reference |  |
| Yes | 0.778 (0.276-2.189) | 0.634 | 0.872 (0.312-2.441) | 0.794 |
| **Therapy** |  | 0.326 |  | 0.155 |
| Radiotherapy | Reference |  | Reference |  |
| Chemoradiotherapy | 0.639 (0.261-1.561) | 0.326 | 0.483 (0.177-1.316) | 0.155 |

**Abbreviations:** Other^a^, American Indian, Alaska Native, Asian, Pacific Islande
