# Supplementary material for: Chemotherapy for locoregionally advanced nasopharyngeal carcinoma: Who really needs it
Source: Cancer Med. 2022 Dec 9;12(6):6994–7004. doi: 10.1002/cam4.5497 (PMC10067101; doi:10.1002/cam4.5497)
Supplement: Supplementary file 8 — Table S8 [file CAM4-12-6994-s006.docx]

**Table S8: Univariate cox analysis of OS and CSS in all stage T3-4N0M0 NPC (N=449)**

| **Variables** | **OS** | | **CSS** | |
| --- | --- | --- | --- | --- |
|  | **HR (95% CI)** | ***P* value** | **HR (95% CI)** | ***P* value** |
| **Age at diagnosis** | 1.041 (1.030-1.052) | **<0.0001** | 1.035 (1.024-1.047) | **<0.0001** |
| **Sex** |  | 0.803 |  | 0.806 |
| Male | Reference |  | Reference |  |
| Female | 0.965 (0.729-1.278) | 0.803 | 1.039 (0.767-1.406) | 0.806 |
| **Race** |  | **0.002** |  | **0.008** |
| White | Reference |  | Reference |  |
| Black | 0.785 (0.524-1.176) | 0.240 | 0.684 (0.426-1.097) | 0.115 |
| Other^a^ | 0.586 (0.434-0.792) | <0.0001 | 0.615 (0.445-0.851) | 0.003 |
| **Marital status** |  | **0.031** |  | **0.033** |
| Married | Reference |  | Reference |  |
| Unmarried | 1.339 (1.027-1.744) | 0.031 | 1.369 (1.025-1.830) | 0.033 |
| **Grade** |  | **<0.0001** |  | **<0.0001** |
| I | Reference |  | Reference |  |
| II | 1.292 (0.639-2.613) | 0.476 | 1.335 (0.632-2.818) | 0.449 |
| III | 0.607 (0.305-1.209) | 0.155 | 0.568 (0.272-1.184) | 0.131 |
| IV | 0.289 (0.137-0.610) | 0.001 | 0.311 (0.141-0.686) | 0.004 |
| **Histology** |  | **<0.0001** |  | **<0.0001** |
| KSCC | Reference |  | Reference |  |
| DNKSCC | 0.437 (0.308-0.621) | <0.0001 | 0.480 (0.328-0.702) | <0.0001 |
| UNKSCC | 0.313 (0.201-0.488) | <0.0001 | 0.357 (0.221-0.575) | <0.0001 |
| Other | 0.463 (0.312-0.688) | <0.0001 | 0.487 (0.316-0.751) | <0.0001 |
| **T stage** |  | 0.174 |  | 0.366 |
| T3 | Reference |  | Reference |  |
| T4 | 1.199 (0.923-1.557) | 0.174 | 1.140 (0.858-1.517) | 0.366 |
| **Surgery to primary site** |  | 0.947 |  | 0.961 |
| No | Reference |  | Reference |  |
| Yes | 0.987 (0.665-1.465) | 0.947 | 1.011 (0.659-1.550) | 0.961 |
| **Radiotherapy** |  | **<0.0001** |  | **<0.0001** |
| No | Reference |  | Reference |  |
| Yes | 0.308 (0.229-0.414) | <0.0001 | 0.274 (0.200-0.376) | <0.0001 |
| **Chemotherapy** |  | **<0.0001** |  | **<0.0001** |
| No | Reference |  | Reference |  |
| Yes | 0.420 (0.318-0.555) | <0.0001 | 0.380 (0.281-0.513) | <0.0001 |

**Abbreviations:** Other^a^, American Indian, Alaska Native, Asian, Pacific Islander.
